# Automated electron diffraction tomography – development and applications

**DOI:** 10.1107/S2052520619006711

**Published:** 2019-08-01

**Authors:** Ute Kolb, Yaşar Krysiak, Sergi Plana-Ruiz

**Affiliations:** a Institut für Anorganische Chemie und Analytische Chemie, Johannes Gutenberg-Universität Mainz, Duesbergweg 10-14, Mainz, 55128, Germany; b Institut für Angewandte Geowissenchaften, Technische Universität Darmstadt, Schnittspahnstrasse 9, Darmstadt, 64287, Germany; c LENS-MIND, Departament d’Enginyeria Electrònica i Biomèdica, Universitat de Barcelona, Martí i Franquès 1, Barcelona, 08028, Spain

**Keywords:** electron diffraction tomography, single-crystal structure analysis, disorder analysis, nanomaterials, electron crystallography

## Abstract

Electron diffraction tomography (EDT) has gained increasing interest, starting with the development of automated electron diffraction tomography (ADT) which enables the collection of three-dimensional electron diffraction data from nano-sized crystals suitable for *ab initio* structure analysis. A basic description of the ADT method, nowadays recognized as a reliable and established method, as well as its special features and general applicability to different transmission electron microscopes is provided. In addition, the usability of ADT for crystal structure analysis of single nano-sized crystals with and without special crystallographic features, such as twinning, modulations and disorder is demonstrated.

## Abstract 2

Electron diffraction tomography, a potential method for structure analysis of nanocrystals, and, in more detail, the strategies to use automated diffraction tomography (ADT) technique are described. Examples of ADT application are discussed according to the material class.

## 1. Introduction   

Crystalline nanomaterials from industrial as well as natural sources are present in nearly every aspect of our live. Their structural characterization over several length scales down to atomic resolution is crucial in order to understand and optimize physical properties and to invent new applications. Diffraction methods using X-ray or neutron sources, are commonly applied for crystal structure analysis of crystalline materials. X-ray powder diffraction (XRPD), a widespread technique for which well consolidated structure analysis routines exist, provides the three-dimensional diffraction space in only one dimension. For large unit cells, low symmetry, phase mixtures and impure samples may cause problems in indexing and correct intensity extraction. Additionally, peak profiles may be broadened and asymmetric due to crystal size, strain effects or disorder. The investigation of single crystals is necessary in order to gain information from the full three-dimensional diffraction space. In contrast to X-rays, which can be used only for structure analysis on single crystals down to about one micron, electrons undergo 10^3^ times stronger interaction with matter but they can be used to obtain individual access to crystals down to few nanometres, so-called nanocrystals. For the investigation of such crystals this is realized in a transmission electron microscope (TEM), providing electron diffraction (ED), atomic-resolved imaging in the conventional mode [high-resolution TEM (HRTEM)] or scanning mode [high-resolution STEM (HR-STEM)] as well as spectroscopic information [energy-dispersive spectroscopy (EDS) or electron energy-loss spectroscopy (EELS)] (Hirsch, 1977 ▸; Reimer & Kohl, 2008 ▸; Williams & Carter, 2008 ▸). One of the main advantages of ED is that data can be obtained with high resolution but smaller electron doses compared to HRTEM or HR-STEM, which means that less radiation damage is produced on the crystal at similar resolution.

ED traditionally uses oriented crystals, providing zonal diffraction patterns (Kolb & Matveeva, 2003 ▸; Gemmi *et al.*, 2003 ▸), which can be envisioned as an approximately planar cut through the diffraction space. Due to simultaneous excitation of many reflections, in-zone diffraction patterns show strong dynamical effects (Gjønnes & Moodie, 1965 ▸; Geuens & Van Dyck, 2003 ▸; Avilov *et al.*, 2007 ▸). In addition, the excitation error causes uncertainties if a reflection is in exact Bragg condition or is cut off-centre by the Ewald sphere (Hirsch, 1977 ▸). Both effects, which change diffraction intensities, thus causing problems in structure analysis, can be reduced by the implementation of electron beam precession (Vincent & Midgley, 1994 ▸; Midgley & Eggeman, 2015 ▸). Cell parameter and space-group determination may be performed based on a set of prominent crystallographic zones usually collected through a tilt of a crystal around a main axis (Morniroli *et al.*, 2007 ▸, 2010 ▸). The crystal orientation is often difficult and time-consuming, thus demanding a well trained operator and an increased exposure time, which can lead to electron beam damage on the sample (Gorelik *et al.*, 2011 ▸). In addition, the collection of only prominent zones results in a significant amount of missing reflections and, which turns out in a low coverage of the diffraction space, and it usually allows only the determination of heavy atom positions (Weirich *et al.*, 2006 ▸).

## 2. Automated diffraction tomography   

In order to gain a robust, reliable and easy applicable method for the acquisition of more complete and kinematic ED data from single nanocrystals, automated diffraction tomography (ADT) was developed (Kolb *et al.*, 2007 ▸). In contrast to the above-mentioned traditional ED methods, ADT utilizes non-oriented (off-zone) ED patterns. The inclination of the electron beam from the zone axis reduces systematic dynamical effects arising from the interaction of systematic reflection classes (such as 00*l*), thus providing diffraction data closer to the kinematical behaviour. As a second benefit, the orientation of a crystal axis along the goniometer axis becomes obsolete and only the adjustment of the eucentric height remains. ED patterns are collected while the crystal is tilted sequentially in fixed tilt steps in the range of 0.2–1° dependent on the goal of the investigation. For diffraction data acquisition, the illumination is set to a nano-sized and semi-parallel beam using a small condenser aperture [nanoelectron diffraction (NED) or nanobeam diffraction (NBD) method according to the TEM manufacturer, but referred hereafter as nanobeam electron diffraction (NBED)] or to a parallel beam that illuminates a larger area of the sample by means of a selected area aperture (SAED method). In respect to data acquisition, this strategy demands the tracking of the crystal during tilt series acquisition by imaging techniques. In general, TEM and STEM imaging can be used equally, but STEM imaging was used for ADT in the first instance due to the lower electron dose applied to the sample. For full integration of the diffraction space wedges, electron beam precession can be applied (Vincent & Midgley, 1994 ▸; Midgley & Eggeman, 2015 ▸). An additional effect of precession electron diffraction (PED) application is the reduction of remaining dynamical effects originating from non-systematic reflections (Oleynikov *et al.*, 2007 ▸). A more detailed description is provided in Section 2.1. In respect to data processing, the first step involves the determination of unit-cell parameters and space groups and, after indexing, in a second step, the extraction of reflection intensities. Off-zone diffraction implies the need for three-dimensional reconstruction of the diffraction space. For this purpose the *ADT3D* program was developed; thus guiding through the different steps in an easy and systematic way (Kolb *et al.*, 2008 ▸; Schlitt *et al.*, 2012 ▸). A more detailed explanation of the *ADT3D* program and its newer version is provided in Section 2.2 (both programs distributed by NanoMEGAS SPRL, Belgium). The logical approach is similarly implemented in other programs (Oleynikov, 2011 ▸; Palatinus, 2011 ▸; Clabbers *et al.*, 2018 ▸). Visual inspection of the total scattering information in three dimensions is particularly important for the detection of additional crystalline individuals inside the investigated volume, as well as special crystallographic features such as superstructures, incoherent modulation, twinning or disorder. Recently, the ADT method proved to be suitable for two-dimensionally disordered samples, which enables *ab initio* structure analysis of the average crystal structure and, subsequently, description of the stacking sequence by means of quantitative comparison of simulated ED patterns with cuts from the measured reciprocal volume (see Section 3).

**Figure 1:**
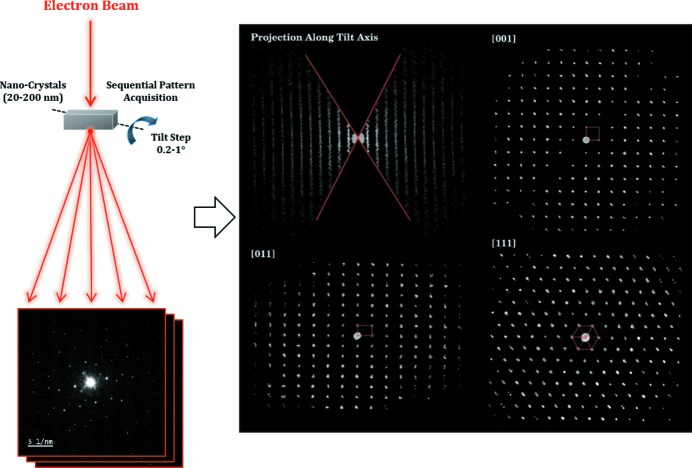
EDT summarized workflow. The diagram on the left-hand side sketches how a stack of diffraction patterns is acquired through a sequential tilt of the nanocrystal under study. The four images from the right-hand side correspond to projections of the reconstructed diffraction space of π-ferrosilicide after data processing (Bowden *et al.*, 2018 ▸).

After the development of ADT in 2007, several groups started to adapt the core idea of ADT; the sampling of the diffraction space via acquisition of non-oriented ED patterns (see Fig. 1 ▸). The developed methods should generally be summarized with the term electron diffraction tomography (EDT), differing only by their applied strategies for data integration and crystal tracking. The choice of the sample illumination method (SAED or NBED) and the application of PED is optional to most of the methods.

In the ADT method, crystal tracking is performed in STEM mode (Kolb *et al.*, 2007 ▸) and the sequential data acquisition is fully automated (Mugnaioli *et al.*, 2009 ▸). Subsequently, successful structure analysis in TEM mode, *i.e.* EDT, with or without PED was demonstrated (Gorelik *et al.*, 2011 ▸). The rotation electron diffraction (RED) method (Zhang *et al.*, 2010 ▸) works in TEM mode in a fully automated way as well. The main difference is that it combines large goniometer tilt steps with small beam tilt for fine sampling of the diffraction space without PED. Another strategy for sampling the reciprocal space is the combination of a continuous crystal tilt with continuous diffraction pattern acquisition, which demands a camera capable of fast acquisition (low read-out time for optimal sampling of the reciprocal space without missing data between acquired frames) and high electron sensitivity. This approach is referred as continuous rotation electron diffraction (Nederlof *et al.*, 2013 ▸) or MicroED (Nannenga *et al.*, 2014 ▸) and it works with an automated and continuous stage tilt. Fast-EDT (Gemmi *et al.*, 2015 ▸) uses a continuous tilt of the stage as well but a crystal tracking based on a preliminary recording of the crystal movement is implemented and, in addition, it couples PED to the continuous crystal tilt for further hardware integration of the reflections. In the case of the continuous rotation electron diffraction (cRED) method (Cichocka *et al.*, 2018 ▸), which follows the same strategy as MicroED, the naming might be misleading because it does not use the strategy of the RED method.

### 2.1. Data acquisition   

The acquisition of diffraction patterns for a tomography data set can be performed using TEM or STEM mode of the microscope. STEM is preferred because it provides an easy and fast way to identify good crystals from their morphology and diffraction pattern quality (Plana-Ruiz *et al.*, 2018 ▸). STEM mode is based on keeping the projector system in diffraction mode, which avoids hysteresis behaviour of the lenses and its consequent variation of the camera length. A focused electron beam, so-called probe, is scanned on a region of interest (ROI) and a digital frame is obtained by means of the intensity integration of the transmitted beam and close-by scattered beams, known as bright-field imaging, or the scattered beams, known as dark-field imaging, for each pixel or beam position in the ROI.

**Figure 2:**
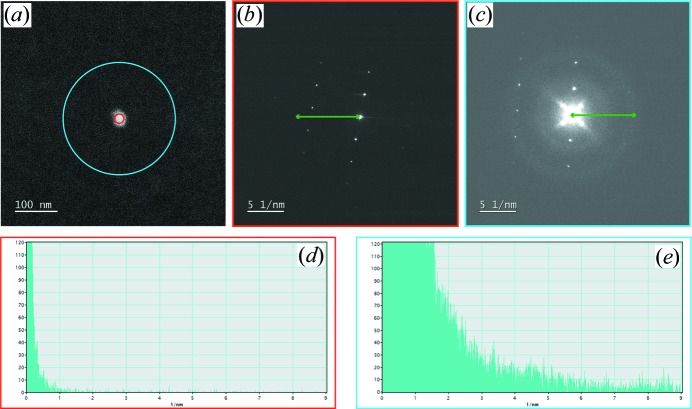
HAADF-STEM image (*a*) showing a single 30 nm × 40 nm WO_3_ particle and diffraction patterns (*b*) and (*c*) corresponding to beam probes of 20 nm [area circled in red in (*a*)] and 200 nm [area circled in blue in (*a*)], respectively. Histograms in (*d*) and (*e*) correspond to the green arrows on (*b*) and (*c*), respectively. Exposure time was set to 0.1 s for (*b*) and 10 s for (*c*) in order to have the same electron dose on the illuminated area.

Another main point for ED analysis is the use of NBED instead of the traditional SAED method. NBED allows spotty diffraction patterns with probe sizes down to the diffraction-limit to be obtained; for instance, around 2 nm probe diameter at full-width at half-maximum with 0.5 mrad of convergence angle if electrons accelerated at 300 kV are considered. When single crystals are sufficiently isolated on the grid, SAED can be used without any problem. However, if a challenging material is structurally investigated with electrons, it is because X-ray diffraction has failed to provide a reliable characterization and/or there are still questions of fine details that remain unanswered. Some examples of such materials may be layered structures such as mullites (Zhao *et al.*, 2017 ▸), single nano-needles from polyphasic powders (Zou *et al.*, 2019 ▸) or nanocrystals embedded in a multiphasic matrix (Nicolopoulos *et al.*, 2018 ▸). In these cases, NBED is the required technique because it gives the possibility of obtaining structural information from only such nanometre-sized areas. The SAED technique may be used to obtain this information but it will be overlapped with diffraction data from other undesired crystal domains, which may make it difficult to properly interpret it. The minimum illuminated area with SAED is limited by the physical aperture inserted in the image plane of the microscope. Nevertheless, Fig. 2 ▸ presents a diffraction pattern analysis that shows how a small beam is still of benefit for isolated nanoparticles. Probes of 20 and 200 nm in diameter were used to acquire diffraction patterns from an isolated WO_3_ particle. The exposure time was set to have the same accumulated electron dose for both patterns. The 20 nm beam allows well resolved reflections with low background to be obtained and without significant contribution of the carbon film. On the other hand, the use of a 200 nm beam also gives well resolved reflections but the contribution of carbon is much higher as more film is illuminated. This is not a problem if ordered materials are studied but it starts to be when diffuse scattering and/or additional weak reflections are considered for investigation.

**Figure 3:**
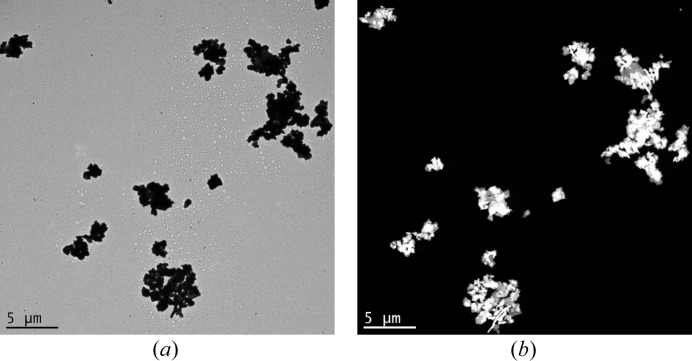
TEM image (*a*) and HAADF-STEM image (*b*) of the same area of BaSO_4_ crystals.

In this context, the logic approach is to use the NBED method in STEM mode. STEM mode coupled with a high-angle annular dark field (HAADF) detector provides better imaging of thinner crystals from an overview of the grid, as it is shown on Fig. 3 ▸. The diffraction contrast is enhanced and finding a good quality crystal becomes easier than in TEM mode.

As the projector system setting is fixed in STEM mode (once the camera length is chosen), the condenser system has to be changed in order to acquire spot-like diffraction patterns. The STEM mode is usually configured with a highly convergent probe for imaging applications. This setting is ideal for atomic resolved imaging because it allows probe sizes below 50 pm to be obtained if aberration correctors are used (Erni *et al.*, 2009 ▸; Barton *et al.*, 2012 ▸; Barthel *et al.*, 2015 ▸), but it is not ideal for diffraction studies as reflections become big disks that overlap each other. Thus, the illumination setting needs to be changed in order to produce a less convergent beam with the disadvantage of getting a bigger probe size. Nevertheless, imaging in STEM mode for diffraction investigations is only needed to identify suitable crystals and reduction of the lateral resolution is not an issue. ThermoFischer (former FEI) TEMs already provide by default such illumination, a so-called microprobe-STEM, but Jeol TEMs do not and they have to be specially aligned. This low-convergent probe STEM mode is referred to in this work as quasi-parallel STEM (QP-STEM) and alignment procedures can be found elsewhere (Yi *et al.*, 2010 ▸; Plana-Ruiz *et al.*, 2018 ▸).

QP-STEM is ideal for diffraction-based experiments according to the following points:

(1) Low-dose imaging with enough contrast to properly identify single-domain crystals (Plana-Ruiz *et al.*, 2018 ▸; Panova *et al.*, 2016 ▸).

(2) Fast crystalline checking. The beam is stopped and the software interface enables the user to position the beam anywhere within the acquired reference image to look at the quality of the diffraction pattern.

(3) Fast check of crystal position in tomography experiments (Kolb *et al.*, 2007 ▸).

Two beam settings are initially saved at the start of the experiment: a full focused configuration for imaging and another one with a tuned probe size for diffraction. The imaging and diffraction settings are here differentiated because the convergence angle is never low enough to have fully focused spots in the diffraction pattern with the minimum probe size. If the condenser system is modified in such a way that the convergence angle is very low (less than 0.1 mrad), the probe size will become too big to obtain accurate crystal images (Yi *et al.*, 2010 ▸). Yet if the convergence angle is kept around 0.5 mrad, an increase of the beam size and a fine adjustment of the diffraction lens results in fully focused reflections without introducing geometrical distortions (an effect produced by the enhancement of the lens aberrations). In the case where the material under study is beam-stable and the unit-cell parameters are not too big to produce overlapped reflections with enough resolution (0.7–1 Å), there is no need to set two different beams. The exposure time of the camera can be increased to have a better signal-to-noise ratio of the reflections and the data can be properly handled.

### 2.2. Data processing and analysis   

**Figure 4:**
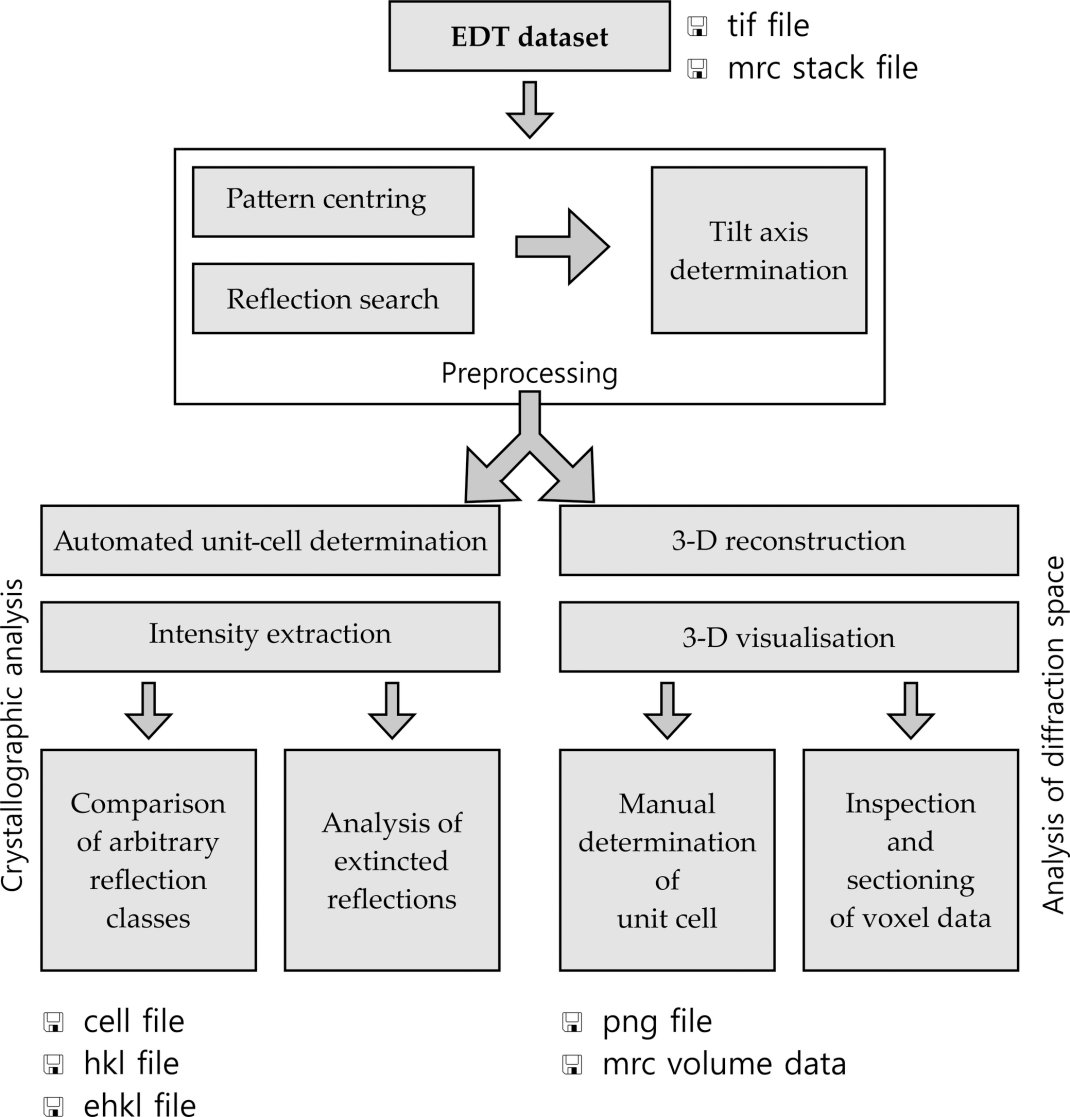
Flow diagram for data processing with *eADT* software.

In order to process and analyse the acquired off-zone ED data sets it is necessary to reconstruct the diffraction space volume, determine the unit-cell parameters, find the possible space groups and extract reflection intensities. The program *ADT3D* was developed in a first step for this purpose, which was followed by an improved version called *eADT*. A detailed description of the basic routines implemented is given by Kolb *et al.* (2012 ▸). Here we outline the changes implemented in *eADT* in comparison to *ADT3D*. The flow diagram presented in Fig. 4 ▸ shows the individual steps for a 3-D reconstruction of the diffraction space and the crystallographic analysis performed by the *eADT* program.

*eADT* was designed with a graphical user-interface that allows the data analysis workflow to be constructed from a variety of modules (parametrized processing objects), which are implemented with the Java object-oriented programming language. The objects, indicated with clearly defined input and output formats, can be assembled freely and quickly according to the intended analysis. As such, *eADT* provides full control on each individual processing step without the use of ‘hidden’ algorithms. This strategy also ensures flexibility because multiple workflows can be setup in parallel. The transparency of the analysis is improved as all generated information such as pattern centres, tilt axis position or 2-D peaks as well as the workflow setup, with or without the data information, can be stored for later use.

The unit-cell determination and intensity extraction are independent of the reconstruction of the voxel space, in contrast to *ADT3D* (Kolb, 2014 ▸). Instead, the extracted peaks can be spanned to a 3-D vector space, which is then used to determine the unit cell. The basis for this reconstruction is the pattern centring, peak extraction and the subsequent tilt axis determination, which are briefly explained below. *eADT* is optimized in such a way that takes advantage of all available PC resources (CPU multithreading for calculations and GPU for visualization), which turns into the fastest processing speed. Therefore, a reconstruction of the voxel space is possible for up to 4 K data sets (requiring 64 GB RAM), including all supporting tools such as volume slicing for symmetry determination or distance and angle measurements in the voxel space.

#### 2.2.1. Pattern centring   

There are two ways to find the centre for each diffraction image of a tilt series. Depending on the beam dose and detector used, a beam blanker may be needed to avoid burning the scintillator. In this case a set of symmetric reflection pairs (Friedel pairs) can be searched. For the case that a measurement could be performed without a beam blanker, the search for the diffraction centre is more straightforward, as it is assumed that the highest intensity with the largest extent corresponds to it. For cases where diffracted intensities are higher than the primary beams, deviations can be manually corrected.

#### 2.2.2. Reflection search   

In principle, high intensities of individual pixels are searched for above a certain threshold value and neighbouring pixels are grouped. *eADT* can determine the minimum values for the intensity threshold and the minimum number of points in a cluster for each diffraction image either automatically or manually. In addition, the shape of a cluster is identified. The ideal shape would be a circle, but small deviations are to be expected. Thus, a smallest possible rectangle is formed around the centre of gravity of each pixel cluster and the intensities are divided by their area. The intensity density can be used as well as a parameter for the determination of true reflections.

#### 2.2.3. Tilt axis determination   

**Figure 5:**
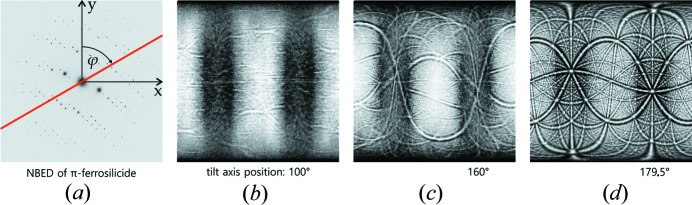
(*a*) Sketch of the reference system used to identify the tilt axis of an ADT measurement. (*b*), (*c*) and (*d*) correspond to three different stereographic projections of different tilt axis positions. (*d*) corresponds to the exact position of the tilt axis, which gives a sharp contrasted image.

The tilt axis position of the ED patterns must be precisely known to enable a three-dimensional reconstruction of the data. It is assumed that the position of the tilt axis is constant during the measurement and the object on which the EDT measurement was performed is a crystalline area. Accordingly, the reconstructed diffraction space of the recorded tilt series should also have periodicities in several directions. For this, the periodicity must be checked as a function of the tilt axis position. The tilt axis position is defined by the angle φ with respect to the *y*-axis of the diffraction pattern and its centre as the origin of a sphere that envelopes the diffraction space. Then, the difference vectors between the individual reflections are projected onto a spherical surface (analogous to Wulff net) and a stereographic projection is calculated. The tilt axis position is selected correctly when sharp lines and points are obtained, as shown in Fig. 5 ▸, and the evaluation is carried out completely automatically, according to an iterative search algorithm (Weirich, 2011 ▸).

#### 2.2.4. 3-D peaks   

*eADT* generates a 3-D matrix data. The 3-D coordinates of the found reflections are calculated by means of the determined tilt axis position, the diffraction image centres and the tilt step through the use of trigonometric functions. As each individual diffraction pattern does not correspond to a plane section of the diffraction space, but is slightly curved (the Ewald sphere), the positions must be back-calculated on a circular surface whose radius depends on the wavelength used.

#### 2.2.5. Unit-cell determination   

The difference vectors of all reflection positions are determined from the 3-D peaks. In the resulting set of position vectors, there are many vectors that point to reflections of similar coordinates. These can be grouped according to three parameters. The user can specify the minimum number of points needed to form a cluster and the maximum distance between points within a cluster. In addition, the maximum distance of the clusters to the origin can be specified. Details about the cluster algorithm can be found elsewhere (Schlitt *et al.*, 2012 ▸).

The calculated difference vector space should be a good approximation of the translation grid from the measured diffraction space. The basis vectors must now be defined so that the position of each significant cluster point can be described by an integer multiple of the basis vectors. *eADT* can automatically find the base vectors through a cluster analysis (Schlitt *et al.*, 2012 ▸), but the result should be checked in the difference vector space and it can be manually corrected if necessary.

## 3. Diffuse scattering   

### 3.1. Introduction   

In addition to dynamic disorder caused by atomic vibrations, static disorder is quite often the case in crystalline materials (Hull & Bacon, 2011 ▸). Consequently, the diffraction space contains information which is not fully describable by a periodic three-dimensional reciprocal lattice. In fact, the relation between defects in real space and diffraction space, shown in Table 1 ▸, is well known (Welberry & Weber, 2016 ▸).

As described in the work by Rozhdestvenskaya *et al.* (2017 ▸), there are several possibilities to handle diffuse scattering for structure determination independent of the used radiation:

(i) non-consideration of the diffuse reflections,

(ii) extraction of intensities at Bragg positions only,

(iii) full analysis of the diffuse scattering.

The Bragg intensity is not sensitive to the short-range order (SRO) of defects. Thus, points (i) and (ii) are usually used to solve a so-called average structure from the scattering data (Zhao *et al.*, 2017 ▸; Krysiak *et al.*, 2018 ▸). On the other hand, the short-range order can be determined by an interpretation of the diffuse scattering between the Bragg reflections, see point (iii). Such an interpretation can be carried out by deriving an analytical model for the studied defect or by simulating disordered crystals and calculating the diffraction pattern from this model. Several groups using X-ray and/or neutron radiation are experts on the simulation of diffuse scattering and analytical description (Aebischer *et al.*, 2006 ▸; Welberry & Weber, 2016 ▸). Even phasing of diffuse scattering using the dual-space method can lead to structural information (Miao *et al.*, 2000 ▸; Ayyer *et al.*, 2016 ▸; Simonov *et al.*, 2017 ▸). In the case of disordered nanocrystals, the use of electrons as the radiation source is unavoidable and the analysis of diffuse scattering becomes more challenging.

For example, the modulated diffuse scattering of vaterite, a polymorph of calcium carbonate, could be analysed and a threefold superstructure was successfully determined *ab initio* using ADT (Mugnaioli, Andrusenko *et al.*, 2012 ▸). However, atomic structure determination of unknown materials by ED becomes difficult if diffuse scattering can no longer be described by a modulation of the reciprocal lattice. In such cases, atomically resolved imaging is indispensable for a reliable structure description because it yields non-periodic object information. In the case of ITQ-39, Wilhammer *et al.* were able to develop a procedure to solve three-dimensional structures of intergrown nanocrystals by a combination of electron crystallography methods; HRTEM imaging through-focal series, RED tomography and crystallographic image processing (Willhammar *et al.*, 2012 ▸; Kapaca *et al.*, 2017 ▸).

### 3.2. Data acquisition and processing   

Krysiak *et al.* (2018 ▸) presented a possible pathway to come closer to the full analysis of diffuse scattering of disordered nanocrystals:

(1) retrieval of the average crystal structure of a material on the basis of EDT data using direct methods,

(2) comparison with structural images produced by electron holography (Chen *et al.*, 2017 ▸),

(3) extraction of experimental diffuse scattering from EDT data,

(4) modelling of disorder and simulation of the total scattering using the *DISCUS* (Proffen & Neder, 1997 ▸) software package,

(5) comparison or refinement on the experimental EDT data.

The third step is highly dependent on the EDT data quality in terms of sampling (tilt step), *d*-value resolution (camera length), signal-to-noise ratio (detector quality) and inelastic scattering (energy filter). The data have to be taken without electron beam precession.

**Figure 6:**
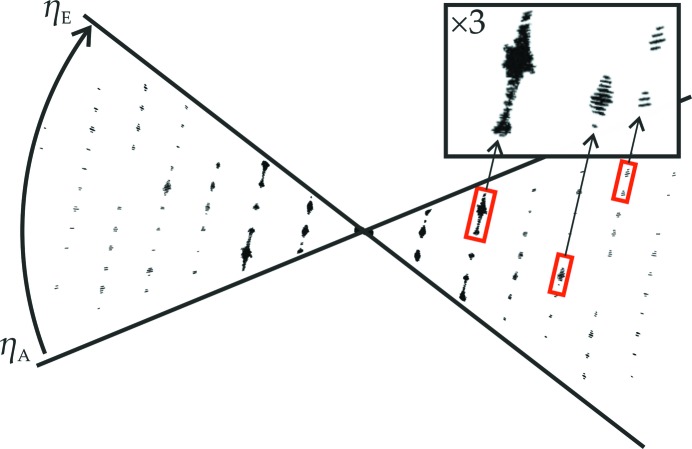
Experimental ED zone of a layer silicate, recorded using ADT and a tilt angle increment of Δη = 1°. Diffuse rods framed in red are shown three times bigger in the black rectangle.

Structures with planar faults usually crystallize as plate-like particles, which leads to preferred orientation. For cases in which diffuse scattering is elongated parallel to the beam direction, its rendering is highly dependent on the tilt step and the distance from the tilt axis, as can be seen in Fig. 6 ▸. In general, it is advisable to use EDT data for the analysis of disorder with the new recording techniques described by Gemmi *et al.* in which a fine sampling of the diffraction space in a short time and low dose (when using direct detection cameras) is possible (Gemmi *et al.*, 2015 ▸; Simancas *et al.*, 2016 ▸).

For the extraction of diffuse scattering, the data has to be reconstructed with a high voxel resolution and the orientation matrix of the average structure has to be defined. This information is, in principle, enough to define planes or pathways of interest in order to project the data in image files and/or text files with pixel positions and related intensities. Therefore, a routine for the extraction of crystallographic zones and diffuse scattering from EDT data was developed.

**Figure 7:**
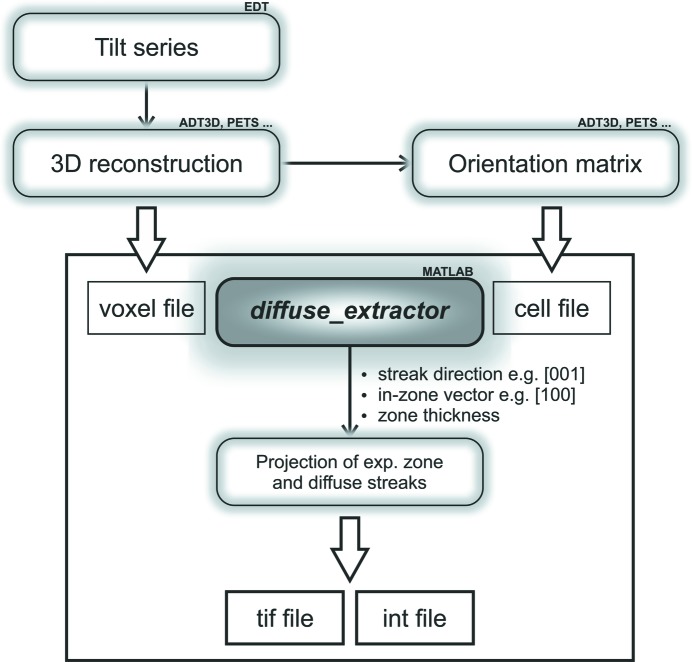
Data flow diagram for the extraction of diffuse scattering from EDT data. The reconstructed EDT tilt series (voxel file) and orientation matrix of the crystal (cell file) are used as data input for the extraction of the diffuse scattering. With the information about the direction of the diffuse scattering, the zone vector and the thickness, the projection(s) of the experimental crystallographic zone and the diffuse scattering along lines are the final output.

**Figure 8:**
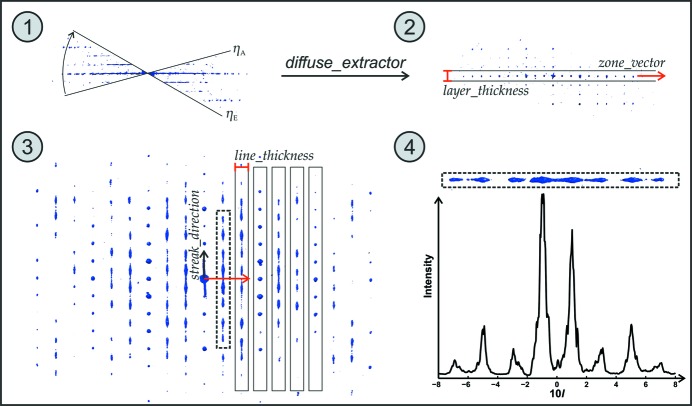
Visualization of the parameters necessary for the extraction of diffuse scattering using the example of zeolite beta: (1) collected tilt series; (2) view along c* with a user-defined zone vector (red arrow) along a* and thickness (in red); (3) calculated projection of the *h*0*l* reflections, respectively [010] zone with the defined streak direction (black arrow) and line thickness of the intensity profiles to extract and (4) calculated projection example for the scattering along pathway 10*l* (from *l* = −8 to +8).

The Matlab-based routine *diffuse_extractor* (see Fig. 7 ▸) allows the automated extraction of diffuse scattering that originates from one- and two-dimensional defects. It only needs the orientation matrix of the lattice and the direction(s) of the diffuse scattering. One example of the extraction of diffuse profile lines from the well known zeolite beta, which tends to have strong intergrowths of different polymorphs, is illustrated in Fig. 8 ▸. (Krysiak *et al.*, 2018 ▸)

One challenge that remains on the analysis of diffuse scattering is how to distinguish it from inelastic scattering. If a comparison is made between energy filtered and non-filtered ED patterns, the difference is that the reflections are sharper for the filtered case (Gemmi & Oleynikov, 2013 ▸; Eggeman *et al.*, 2013 ▸); thus, the contribution from the inelastic scattering is the broadening of the acquired reflections. In the case of disorder, the diffuse scattering will become broader as well. However, when streaks are observed on the diffraction patterns, this can be quantitatively differentiated because the disorder effect causes diffuse lines along determined directions while the inelastic scattering is isotropic. On the other hand, if the disorder is presented in 2-D planes, the only way to quantitatively differentiate both contributions is the use of an energy filter. It is worth saying that the difficulty when trying to differentiate both scattering types increases as the atomic number of the material decreases because of an increased inelastic scattering probability.

### 3.3. Modelling and simulations   

The aforementioned data can be taken into account for a comparison with simulated total scattering. The simulation of total scattering is naturally dependent on the used model. For an automated comparison of experimental and simulated scattering data, it is very helpful if the modelling can be designed according to SRO parameters. Some computer programs for diffraction image simulation have been established over the last 20 years (Proffen & Neder, 1997 ▸; Goossens *et al.*, 2011 ▸; Refson, 2000 ▸; Treacy *et al.*, 1991 ▸; Tucker *et al.*, 2007 ▸). *DISCUS* (Proffen & Neder, 1997 ▸) especially provides a wide range of possibilities to approach a real structural description. The program allows a wide range of disorder models at atomic resolution to be simulated, the corresponding single-crystal or powder diffraction data (X-ray, neutron, ED) to be calculated and the model to be adapted to the corresponding experiment by reverse Monte Carlo (RMC) (McGreevy & Pusztai, 1988 ▸) and/or an evolutionary algorithm. Likewise, the pair distribution function analysis (PDF) (Warren *et al.*, 1936 ▸) is readily calculated.

In the case of zeolite beta the extracted diffuse profile lines fit best to a polymorph mixture BEA/BEB = 47 (3):53 (3), which is in good agreement with powder simulations, where a composition of BEA/BEB = 50:50 was determined for the synthesized powdered sample (Krysiak *et al.*, 2018 ▸).

## 4. EDT application   

EDT is nowadays used by a growing number of scientific groups. The broad applicability of EDT covers inorganic materials such as alloys (Bowden *et al.*, 2018 ▸), natural minerals (Gemmi *et al.*, 2012 ▸; Rozhdestvenskaya *et al.*, 2017 ▸; Németh *et al.*, 2018 ▸), archaeological materials (Zacharias *et al.*, 2018 ▸; Nicolopoulos *et al.*, 2018 ▸), a large number of zeolites (Jiang *et al.*, 2011 ▸; Willhammar *et al.*, 2012 ▸; Mugnaioli & Kolb, 2014 ▸; Bereciartua *et al.*, 2017 ▸), phosphates (Mugnaioli, Sedlmaier *et al.*, 2012 ▸), perovskites (Klein, 2011 ▸; Gorelik *et al.*, 2011 ▸), samples with organic ligands (Förster *et al.*, 2015 ▸), zeolite with incorporated templates (Rius *et al.*, 2013 ▸) or hybrid materials (Bellussi *et al.*, 2012 ▸), as well as more electron beam-sensitive samples such as metal–organic frameworks (MOFs) (Denysenko *et al.*, 2011 ▸; Rhauderwiek *et al.*, 2018 ▸; Wang *et al.*, 2018 ▸), small organic molecules (Gorelik *et al.*, 2012 ▸) such as pigments (Teteruk *et al.*, 2014 ▸) or pharmaceuticals (van Genderen *et al.*, 2016 ▸; Wang *et al.*, 2017 ▸; Palatinus *et al.*, 2017 ▸; Das *et al.*, 2018 ▸; Gruene *et al.*, 2018 ▸; Jones *et al.*, 2018 ▸) as well as proteins (Nederlof *et al.*, 2013 ▸; Nannenga & Gonen, 2014 ▸; Lanza *et al.*, 2019 ▸). Furthermore, the method was proven to be even suitable for *in situ* investigations (Karakulina *et al.*, 2018 ▸). The crystal sizes shown here in these examples are in the regime of several hundred down to one hundred nanometres.

### 4.1. Small crystals investigated with ADT   

As described above, the optimal method for the investigation of nanocrystals, in the regime of a few tens of nanometres, is the use of ADT in STEM mode combined with PED. These tiny crystals may be domains of a larger crystals, intermediates grown in early nucleation states or even single phases embedded in a different phase matrix. Moreover, they usually are agglomerated. A successful structure analysis was performed from a phase mixture of the known ZnSb phase strongly agglomerated with a new phase of Zn_1−δ_Sb (Birkel *et al.*, 2010 ▸). Two orthogonal tilt series from the same 50 nm crystal were necessary to gain enough data suitable for structure analysis in space group 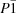, which has been necessary due to pseudosymmetry. In the case of charoite, a rock-forming mineral from the Murun massif in Yakutia (Russia), fine fibres of polytypes share a common ***c***-axis. In order to avoid intensity overlap originating from different polytypes, the ADT technique with a beam of 70 nm diameter was used (Rozhdestvenskaya *et al.*, 2010 ▸). Another example is the structure solution of a poly(triazine imide) with incorporated LiCl used for photocatalytic reactions (Mesch *et al.*, 2016 ▸). This material crystallizes in platelets of about 50 nm in lateral dimension growing in a stacking way because of its layered structure. In order to obtain ADT data suitable for the refinement of the H/Li distribution, very thin platelets had to be selected which were only visible in the high contrast of STEM imaging. Boron oxynitride prepared under high pressure at 1900°C (Bhat *et al.*, 2015 ▸) is another example of crystals with a size of around 50 nm. The achieved data quality allowed the refinement of the solved structure in space group *R*3*m* with two different rhombohedral settings (obverse and reverse) of the hexagonal cell, which revealed twin domains on the crystals. An alternative strategy for the structure analysis of twinned crystals is the direct address of one twin domain, as performed with a triple twin of a Bi(MOF) (Feyand *et al.*, 2012 ▸). Disorder analysis was performed on ∼30 nm tips of crystalline needles of pigment red (PR170) (Teteruk *et al.*, 2014 ▸); further examples are provided in Section 4.4. A full crystal structure solution of nucleation intermediates was possible from even smaller crystals as described in the following section.

### 4.2. Biominerals solved using ADT   

**Figure 9:**
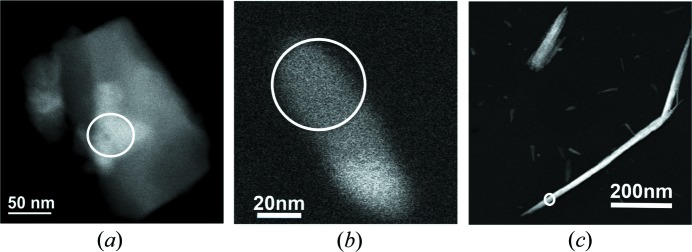
STEM images of hy­droxy­apatite (*a*), vaterite (*b*) and calcium carbonate hemihydrate (*c*). Electron beam diameters used for ADT are indicated with white circles.

In the broad class of biominerals, carbonates and phosphates are amongst the most thoroughly investigated materials, but still reveal surprises such as the discovery of a new hydrated calcium carbonate phase, apart from the known hydrated crystal phases mono­hydro­calcite (MHC: CaCO_3_·H_2_O) and ikaite (CaCO_3_·6H_2_O) (Zou *et al.*, 2019 ▸). The new calcium carbonate hemihydrate (CaCO_3_·0.5H_2_O) has been detected in the course of the magnesium-assisted crystallization of amorphous calcium carbonate. The crystals, which gradually transform to MHC in solution but they are stable in vacuum for a few months, exhibit thin needles with tips of approximately 20 nm as shown in Fig. 9 ▸. The structure was solved based on ADT data using a 50 nm beam from a mixture of several phases and clearly defined including the water position. Larger but comparable needles have been detected for a new calcium carbonate phase, called monoclinic aragonite (Németh *et al.*, 2018 ▸). This cave mineral, analysed using EDT, exhibits needles of several microns in length narrowing to a tip of some tens of nanometres. The needles exhibit strong diffuse scattering, which hampers structure analysis. In an earlier investigation, ADT was used to solve the long-lasting quest of the vaterite structure on crystals of 40 nm × 20 nm size (see Fig. 9 ▸), and to quantitatively derive, in addition to the monoclinic average structure, the superstructure according to the threefold expansion of the ***c***-axis (Mugnaioli, Andrusenko *et al.*, 2012 ▸). Fig. 9 ▸ provides a comparison of the STEM images of crystals used to determine the noncentrosymmetric space group of human tooth hy­droxy­apatite (Mugnaioli *et al.*, 2014 ▸).

### 4.3. Small organic molecules solved by ADT   

ADT in STEM mode has been used, since the development of the method, for *ab initio* structure solution of small organic molecules (Kolb *et al.*, 2010 ▸). The size of the investigated crystals is usually in the regime of hundreds of nanometres and an electron beam dimension of approximately 50 to 100 nm is chosen. In order to perform a successful crystal structure analysis, the acquired diffraction data needs to be of high completeness to avoid strong deviations in atomic positions, thus leading to distortions of the molecular structure. ADT data resolution for organic molecules ranges from around 0.6–0.8 Å, and it is usually limited to 1.0 Å for structure solution but fully used in subsequent refinement routines. In the refinement step, a higher quality of electron diffraction data, meaning less radiation damage in the case of beam-sensitive materials, improves significantly the final residual values (Kolb *et al.*, 2010 ▸). In Kolb *et al.* (2010 ▸), a full structure solution using *ADT* [1k CCD (Gatan 794MSC), @RT, tomography sample holder (Fischione), PED (NanoMEGAS DigiStar)] was successfully performed on 9,9′-bianthracene-10-carbo­nitrile (CNBA), a non-linear optically active compound, and compared to the traditional in-zone electron diffraction approach. In spite of the high residuals, mainly caused by beam damage, all structures could be determined with maximum atomic position deviations of 0.2 Å when compared to the structure derived by X-ray single crystal analysis. The crystals used for the structural investigation of tri- and tetra-*p*-benzamides (OPBA3 and OPBA4) were in the regime of a few hundreds of nanometres which are much smaller than the CNBA crystals (Gorelik *et al.*, 2012 ▸).

**Figure 10:**
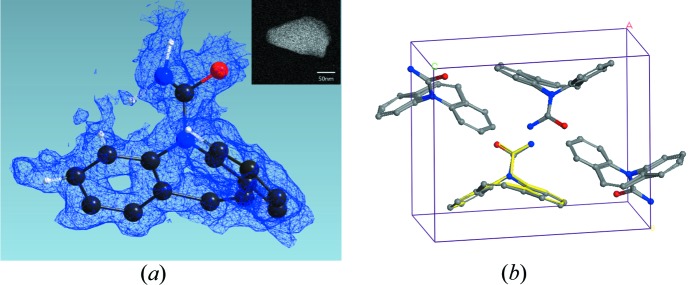
(*a*) Molecular geometry of CBMZ with the observed scattering potential (including detected maxima close to expected hydrogen positions indicated in white), crystal image (inset) used for ADT data acquisition and sketch of the molecule (left side). (*b*) Comparison of the CBMZ crystal structure without hydrogen atoms with the published structure (in yellow). Only one molecule for the published structure is shown.

For more beam-sensitive samples, such as pharmaceuticals, EDT data should be acquired upon sample cooling with liquid N_2_. ADT data acquisition on carbamazepine (CBMZ) nanocrystals, as shown in the inset of Fig. 10 ▸(*a*), were performed using a cryo-transfer tomography holder (Fischione), a 4k CCD camera (Gatan US4000) and a beam precession unit (NanoMEGAS DigiStar). The crystal was 150 nm × 50 nm in size, thus it did not allow a large movement of the 50 nm beam over the sample for further beam damage reduction. The ADT acquisition took approximately 10 min to collect 97 diffraction patterns covering a tilt range between −44° to 52°. After data processing, cell parameters (*a* = 7.49 Å, *b* = 11.28 Å, *c* = 13.60 Å, α = 90.2°, β = 93.1°, γ = 90.5°; usual uncertainty range 1–2% for cell axis and 1° for cell angles) and space group *P*2_1_/*n* of form III were confirmed. Data extraction delivered 5495 reflections up to 0.6 Å resolution. The first 18 potential solutions, derived from structure solution with *SIR14* (Burla *et al.*, 2015 ▸) at 0.8 Å resolution, resembled all carbon, nitro­gen and oxygen positions of carbamazepine. Structure refinement performed in *SHELXL* (Sheldrick, 1997 ▸, 2015 ▸) with the full resolution range was stable and six additional maxima were indicated close to the expected hydrogen positions (see Fig. 10 ▸ *a*). An unconstrained refinement of the CBMZ structure without hydrogens resulted in a residual value of *R*_1_ = 43.6%. If the hydrogens are refined in riding mode (overall *U* ^*ij*^ = 0.133 Å^2^), the residual increases to *R*_1_ = 43.9% for *F*(*hkl*) > 4σ_*F*_ and 49.9% for all acquired data. Table 2 ▸ summarizes the structure solution parameters for CNBA, OPBA3 and CBMZ, including the structures already solved by EDT with a direct detection device; CBMZ1 (van Genderen *et al.*, 2016 ▸) and CBMZ2 (Jones *et al.*, 2018 ▸).

### 4.4. Structure solution from disordered nanocrystals using ADT   

The structure solution of disordered nanocrystals was until now solved via the two approaches referred to by Rozhdestvenskaya *et al.* (2017 ▸), which were described in Section 3.1.

In the case of aluminium borate (Zhao *et al.*, 2017 ▸), the average structure could be solved by not taking into account additional diffuse reflections. Then, the average structure served as the basis for modelling a disordered *AAB·AAB·AAB*… stacking sequence, whose diffraction simulation could be compared qualitatively with the ADT data. The use of a small electron beam (around 50 nm) was essential because ordered and disordered domains occurred within a single crystal. However, there are also examples in which the consideration of diffuse Bragg reflections could lead to an average structure, such as in the analysis of sodium titanate nanorods (Andrusenko *et al.*, 2011 ▸). These materials are typically affected by pervasive defects, such as mutual layer shifts that produce diffraction streaks along c*, which could made be visible by HRTEM imaging. Another example in which the consideration of diffuse Bragg reflections led to an *ab initio* structural model, which takes into account the average modulation, is the CaCO_3_ modification vaterite (Mugnaioli, Andrusenko *et al.*, 2012 ▸). The small crystal size of below 50 nm here was one of the big challenges, besides the stacking disorder. A much more complex structure (86 independent atoms in the asymmetric unit), the mineral denisovite, could be determined by the same procedure as the examples described above (Rozhdestvenskaya *et al.*, 2017 ▸).

In order to be able to perform the ‘full analysis of the diffuse scattering’ of a further unknown material, an average structure model is essential in addition to the disorder modelling and simulation of the corresponding total scattering. For the example of zeolite beta, which was already extensively discussed in Section 3, it was shown how, despite strong intergrowths, the structure of two polymorphs could be successfully solved on the basis of just one ADT data set (Krysiak *et al.*, 2018 ▸).

## 5. Conclusion   

The high potential of EDT for crystal structure analysis of nanocrystals, where other diffraction methods such as X-ray single crystal or powder diffraction fail, has been demonstrated many times since 2007, when the development of the first method was described. Here we provide a description of the different approaches and propose to reference all the different acquisition techniques in the general name of EDT. Apart from this survey, we focus on the ADT method developed in our group, which is so far the only method combining STEM imaging for tracking purposes with tomographic diffraction data acquisition. The benefit of this approach is discussed from a technical point of view as well as demonstrated in the case of applications to several classes of materials. This includes the accessibility of crystals down to a size of 20 nm and the possibility of accessing the average structure of the disordered material with a subsequent description of the disorder. In addition, it was demonstrated that ADT is capable of acquiring EDT data from beam-sensitive materials with only weak scattering atoms by using a CCD camera. Especially for phase mixtures, it is important to be able to derive information from all phases, which may not only differ in terms of crystal size but as well by properties such as crystallinity, solubility, stability and bioavailability. As an example, a structure analysis of carbamazepine was successfully performed based on a single ADT data set. The structure, which was refined without any constraint, is in good agreement with those solved by EDT on the basis of significantly larger crystals.

Furthermore, it is demonstrated that ADT provides a high accessibility to structurally complex samples. This is increasingly important for successful tailoring of physical properties for a wide range of applications. The majority of materials used in this respect contain crystalline nano-sized domains, in many cases bearing defects, which drive the desired physical effects. The accuracy of the STEM imaging used in ADT is ideal to access and characterize one by one these single domains.

Beside the large number of materials which have been structurally determined solemnly on the basis of EDT data, the method provides a strong potential to be combined to complementary techniques such high-resolution imaging (HR-TEM/HR-STEM), diffraction methods that provide bulk information, such as XRPD and NMR, as well as to simulation approaches.

## Supplementary Material

Crystal structure: contains datablock(s) I. DOI: 10.1107/S2052520619006711/je5012sup1.cif

Structure factors: contains datablock(s) I. DOI: 10.1107/S2052520619006711/je5012Isup2.hkl

CCDC reference: 1915324

## Figures and Tables

**Table 1:** Relation between crystallographic defects and diffuse scattering in diffraction space

Dimension of the defects	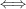	Type of diffuse scattering
0-D point defects		3-D undefined and anisotropy
1-D line defects		2-D planes ⊥ lines in real space
2-D planar defects		1-D stripes ⊥ planes in real space
3-D bulk defects		0-D additional intensities

**Table 2:** Structure solution and refinement based on ADT data for 9,9′-bianthracene-10-carbo­nitrile (CNBA), tri-*p*-benzamide (OPBA3) and carbamazepine (CBMZ) CBMZ1 and CBMZ2 are solved structures using EDT from van Genderen *et al.* (2016 ▸) using a Timepix ASIC detector (software: Amsterdam scientific instruments, Netherlands) and Jones *et al.* (2018 ▸) using Ceta CMOS (Thermo Fisher) detector. Measurement conditions indicate the use of sample cooling (RT: room temperature; LT: liquid nitro­gen cooling) and the used detector.

Sample	Chemical formula	Space group	Lateral crystal size (nm)	Measurement conditions	Tilt range (°)	No. of reflections measured, independent	Completeness (%)	*R*_int_, *R*_solution_ (@res)†	*R*_1_(@res)‡
CNBA	C_29_N_1_	*P*2_1_/*c*	700 × 700	RT CCD	120	9415, 3519	87	21.8, 24.4 (8)	39.0 (8)
OPBA3	C_18_O_6_N_3_H_15_	*P*2_1_/*c*	500 × 1000	RT CCD	120	9185, 3078	81	34.6 (10)	58.0 (10)
CBMZ	C_15_N_2_OH_12_	*P*2_1_/*n*	150 × 100	LT CCD	97	5495, 3588	89	15.9, 26.8 (8)	43.6 (6)
CBMZ1	C_15_N_2_OH_12_	*P*2_1_/*n*	1200 × 800	RT ASIC	51	2202, 1071	45	35.8, – (0.8)	28
CBMZ2	C_15_N_2_OH_12_	*P*2_1_/*n*	700 × 1600	LT CMOS	140	4682, 1044	88	19.5, – (1.0)	19.3

† Burla *et al.* (2015 ▸).

‡ Sheldrick (1997 ▸, 2015 ▸).
